# What Helps Oiled Wildlife Responders Care for Animals While Minimizing Stress and Compassion Fatigue

**DOI:** 10.3390/ani11071952

**Published:** 2021-06-30

**Authors:** Polly Yeung, Bridey White, Michael Ziccardi, B. Louise Chilvers

**Affiliations:** 1 School of Social Work, Massey University, Palmerston North 4442, New Zealand; 2 Wildbase, School of Veterinary Science, Massey University, Palmerston North 4442, New Zealand; b.j.white@massey.ac.nz (B.W.); b.l.chilvers@massey.ac.nz (B.L.C.); 3 One Health Institute, University of California, Davis, CA 95616, USA; mhziccardi@ucdavis.edu

**Keywords:** compassion satisfaction, compassion fatigue, burnout, resilience, professional quality of life, oiled wildlife responders, oil spills

## Abstract

**Simple Summary:**

An oil spill can have severe environmental effects, as well as impacting the wellbeing of first responders. Oiled wildlife responders are a key professional group for the identification of wildlife at risk and to provide measures to rescue, rehabilitate and release wildlife back to their clean habitat. Currently, there is limited information documenting impacts to responders’ mental health during a spill response relating to stress, burnout and compassion fatigue; thus, there are limited interventions and strategies that can support responders and address these issues. A survey for oiled wildlife responders who participated in either the New Zealand MV Rena incident or the US Refugio pipeline oil spill was instigated to investigate and contribute to this knowledge gap. Results indicated that to support the health and wellbeing of oiled wildlife responders and sustain them to provide the best achievable care for oiled wildlife, ongoing professional training supported by organizations and professional networks in the areas of emergency preparedness, resilience, self-care and capacity building should be made available to enhance compassion satisfaction and role fulfilment.

**Abstract:**

Oil spills are environmental disasters and their long-term impact is not just a concern for the environment and economy, but also for first responders’ health and wellbeing. Wildlife, such as aquatic birds and certain marine mammals, are highly susceptible to physiological effects of oiling, and oiled wildlife responders are crucial to provide measures for their survival. The purpose of this research was to explore the experiences of oiled wildlife responders and what factors and conditions have helped or inhibited the responders to care-affected wildlife. This study collected responses (*n* = 50) from a survey of responders who attended either the New Zealand MV Rena or US Refugio pipeline oil spills. Study participants were mostly older (>40), highly educated females. We found there were significant differences in compassion satisfaction, resilience, burnout and overall satisfaction based on age, gender and role. While most responders have only attended limited numbers of oil spill incidents, they reported positive experiences and found it rewarding. Findings from responders indicated that to lessen stress and compassion fatigue during an incident, provision of training and support from professional organizations equips responders with knowledge and skills that can support their personal resilience to respond to disaster events.

## 1. Introduction

Oil spills are environmental disasters that often lead to negative and long-term impacts on the environment. In the past century, more than 7 million tonnes of oil have been spilled and caused severe impacts on the environment [1]. More recently the Deepwater Horizon Oil spill (the BP oil spill) in 2010 claimed as one of the largest oil spills in international history, with more than 800 thousand tonnes of crude oil spilled [2,3]. Tens of thousands of workers and volunteers responded to aid in clean-up activities of these spills. The long-term impact of environment disasters, such as oil spills, is not just a concern for the economy, ecology, and environment of an area, it can also affect the health and wellbeing of people and their community [4]. Despite the significant physical and emotional health risks of oil spills, D’Andrea and Reddy [5] have argued that only a few studies have attempted to assess the human health and psychological wellbeing from more than 40 oil spill disasters occurred around the world. These articles have reported increased risks of physical and mental health symptoms following an oil spill exposure such as headache, fatigue, skin rash, depression, anxiety, and posttraumatic stress syndrome [6,7,8,9,10,11]. Although mental health after the oil spill was reported as one of the key affected areas for human beings, impacts documented varied substantially with some reports of residents in the oil spill areas being more at risk if they were physically exposed to oil or were financially impacted [12,13]. Other studies reported that individuals who participated in oil spill response and clean-up activities were also potentially a high-risk group, as exposure to hazardous chemical, and stress of response can induce adverse mental health effects [9,14]. Increases in anxiety were also found among people residing in places affected by oil spill [15,16].

The occurrence of larger scale disasters means, aside from emergency first responders (e.g., firefighters), more clean-up and recovery workers are and will be required to work along with volunteers and workers from other social and industrial sectors [17]. Although there is a wealth of research on trauma-exposed populations after disasters, much of it has focused on individuals rather than occupational groups [18]. In the area of oil spills, a small portion of studies have reported potential health risks of oil spills on safety of workers, toxic effects in first responders, workers, volunteers and community members in clean-up activities, and psychosocial and ecosystem effects on human health [2,8,19,20,21,22,23,24]. Brooks et al. [25] have argued that factors such as training length and timing of deployment; traumatic exposure; emotional involvement; leadership; inter-agency co-operation; social and formal support; role clarity; job demands; perception of safety; coping skills; and personal and professional growth could affect psychological risk and resilience during and after disasters among different occupations. With the public expectation of disaster response becoming more prevalent, organisations need to consider their degree of disaster preparedness and impacts on their staff wellbeing and productivity [26].

Despite the growing concerns of traumatic stress within disaster-exposed occupations, most of the attention tends to focus on firefighters, police officers, healthcare workers, emergency medical technicians and others involved in human search and rescue operations [27] with very limited mention of oiled wildlife responders. One of the most noticeable and widely scrutinised effects of oil pollution, particularly during acute oil spills, are the effects on wildlife and the environment [28,29]. Oil affects wildlife directly and indirectly and if oiled significantly, wildlife will often die in the absence of human intervention [30]. During such maritime environmental emergencies, oiled wildlife responders are commonly mobilised into the field to identify wildlife at risk, provide protection measures for wildlife and/or to respond to impacted wildlife [31]. As oiled wildlife response has developed into a multi-disciplinary professional effort since the mid-90s, oiled wildlife responses are becoming better coordinated to develop different response models for better practice guidelines [32]. Any oiled wildlife response not only requires a number of key components such as a place to operate from, and appropriate equipment, but they also need wildlife personnel such as oiled wildlife responders in field and rehabilitation facilities operations [33]. Although there is good evidence of successful rehabilitation following oiling of seabirds [34], the question of whether or not oiled wildlife should be rescued and rehabilitated is not without its debate and controversy. The issue becomes particularly apparent after a significant oil spill such as the Deepwater Horizon incident, in which questions were raised as to whether such effort and financial resources should have been used to save, rehabilitate and release affected wildlife [29,30,35]. One of the critical factors identified as affecting the success of an oiled wildlife is the timely collection and rehabilitation of oiled animals by trained and experienced personnel [33]. Not only can oiled wildlife responders be exposed to a range of physical (e.g., transportation processes, working in extreme climate, the use of untrained personnel), chemical (e.g., pollutants from the incident) and biological hazards (e.g., zoonotic diseases from working wildlife), there is also a risk for mental health, coming from the trauma of working with large numbers of sick, injured and dying animals [31]. A considerable amount of research has reported that animal euthanasia is one of the factors that creates distress and contributes to the incidence of mental health issues in professionals who work with animals [36,37,38]. In addition to dealing with stress and fatigue in oil spill response, oiled wildlife responders will need to be aware of the high expectations of public and the role of media in affecting the public image of a response [39,40].

Aside from having official responders managing operational and mechanical processes during the spills, members of local communities and other volunteers have been shown to play a significant role in helping with oiled wildlife response and clean-up [41,42]. However, the use of volunteers is not always straightforward as Spears et al. [43] and Clumpner [44] both reported that volunteers often “self deploy” and arrive in large numbers and are usually untrained in the mechanisms to deal with issues such as oil spill response. Hur [45] also reported that following an oil spill in Korea in 2007, authorities and infrastructure were overwhelmed by thousands of volunteers and resulted in unclear responsibilities, poor organisation of resources and harm to volunteers due to ineffective coordination. Yeung et al. [46] argued that the extensive usage of volunteers in a disaster event calls for more careful examination of their experience in the field, including training programmes, supervision and stress and burnout prevention. Mental health and overall wellbeing specific to oiled wildlife responders is an emerging topic but also often one that is neglected as a key readiness activity. Gibson [47] argued that these issues can become an afterthought once the impact has already become apparent and there may not be follow-ups to address these issues before the next incident. Even when information on stress, burnout, and compassion fatigue to animal-care professionals and volunteers are acknowledged, they are historically integrated into health and safety teaching only as a small component [48,49].

Professionals that work in disasters and with victims of trauma, including those in animal and wildlife care, are at risk of experiencing burnout and compassion fatigue [46]. Compassion fatigue (CF) is defined as “a state of exhaustion and dysfunction—biologically, psychologically, and socially—as a result of prolonged exposure to compassion stress and all that it evokes” [50] (p. 253). Burn out (BO) refers to a state of mental, emotional, and physical exhaustion caused by prolonged emotional stress on the job, which can lead to feelings of helplessness and difficulty in dealing with work and everyday lives [51,52]. However, providing support, care, and empathy in rescue, recovery, and rehabilitation can enrich people’s lives and this enrichment is termed as compassion satisfaction (CS), which measures affirmative experiences [52,53]. CS is believed to buffer the negative effects of BO and CF, and meanwhile resilience (RS) has been found to play a similar role due to its ability to adapt and maintain health, both psychologically and physically [54]. RS is defined as an individual’s ability to effectively adapt and cope with challenging situations that can act as a counterbalance to stressors [55,56]. The quality of and capacity to provide animal and wildlife care are directly affected by professionals’ skills and their own wellbeing. The stress and impact on mental health during and after incidents are important to address. If overlooked or not properly managed, such stressors can affect emotional and physical health, and this can have flow-on effects for retaining professionals in this specific workforce for future effective operations.

The current study focused on oiled wildlife responders who participated in either the New Zealand (NZ) MV Rena and United States (US) Refugio pipeline oil spills. On the 5 October 2011, the MV Rena grounded 12 km off the coast of the Bay of Plenty, NZ, resulting in the spilling approximately 350 tonnes of heavy fuel oil. The Refugio pipeline oil spill occurred on the 19 May 2015 and released 142,800 US gallons (540 tonnes) of heavy fuel oil into the area of the Refugio State Beach, California, United States. This study aimed to explore the experiences of oiled wildlife responders from these spills and what factors and conditions have helped or inhibited the spill management team to rescue, recover and care for the wildlife animals. It also focused on investigating these responders’ health and wellbeing status through understanding their professional quality of life, perceived effects of helping in oil spills, resilience, and role/life satisfaction. This understanding will help develop strategies that can enhance oiled wildlife respondents to be more effective and efficient in their roles and to minimise the damaging effects of oil pollution on wildlife in the future.

## 2. Materials and Methods

A cross-sectional questionnaire design, using an anonymous online survey powered by Survey Monkey, was chosen as it allowed for the collection of data from a wide range of respondents. A self-selected, purposive sample was used. An email containing information about the study and the online questionnaire link was sent out to the network of oiled wildlife responders who were mobilised either by Maritime NZ (NZ central government department responsible for marine oil spill response in NZ) or Wildbase, Massey University, Aotoearoa NZ (a professional oiled wildlife response team contracted to the NZ central government) for the NZ MV Rena spill or by the Oiled Wildlife Care Network (OWCN), University of California at Davis [a professional oiled wildlife response team contracted by California Department of Fish and Wildlife’s Office of Spill Prevention and Response (OSPR)] for the Refugio spill.

Participants who had participated in the NZ MV Rena or US Refugio wildlife responses were eligible to complete the questionnaire. Implied consent was obtained through clicking the submission button at the end of the questionnaire. Ethics approval was gained from Massey University Human Ethics Committee: Southern B (18/54) prior to the commencement of the research. Sixty-two responses were retrieved from the online survey portal. After data cleaning and mining, 12 responses were omitted due to significant data missing (e.g., 50% or more) from the main questions and the socio-demographic information. A total of 50 responses were retained for analyses. Over half of the responders (68.0%, *n* = 34) participated in the NZ MV Rena spill, with the remaining 32.0% (*n* = 16) in the US Refugio spill. Given the online survey was circulated to many different groups and professional networks, it was impossible to ascertain the actual response rate.

The survey consisted of the following sections:

Involvement with previous oil spill incidents. Respondents were asked to provide information of their past involvement in terms of (1) which incidents; (2) number of overall spills attended; (3) who initially activated the mission; (4) their roles and involvement; and (6) clarity of position, tasks, responsibility, and training.

Overall experience of responding to incidents. Participants were asked to reflect on the period they were involved in the spill, skills being utilised, and the amount of workload, update and support received. They were also asked how rewarding the experience was in responding to the oil spill, using a 5-point Likert scale from “1 = not at all rewarding” to “5 = definitely rewarding”. They were also asked if they would participate in oil spill response again in the future with “1 = not at all” to “4 = yes, definitely”.

Perceived effect of helping in oil spill. Participants were asked to rate the extent to which three aspects of their lives had changed as a result of helping in oil spills: finances, social relationships, and physical health based on the work by Drescher et al. [2]. Participants responded to these items using a 5-point Likert scale from “1 = greatly worsened” to “5 = greatly improved”. These questions were reverse scored and averaged for an overall general effect score to make interpretation simpler for analyses as per Drescher et al.’s [2] suggestion. Higher the score means their health and wellbeing have worsened. In addition, from these data, two groupings were created for descriptive analysis purpose [57]. Ratings were collapsed for each dimension into two categories to indicate worsening (score of 4 or more on the reversed scale) or no change/improvement (score of 3 or less). Cronbach’s alpha was reported at 0.81.

Professional quality of life (ProQOL). Participants were asked to respond to ProQOL, which consists of three subscales to assess Compassion Satisfaction (CS) of 10 items, Compassion Fatigue (CF) of 10 items, and Burnout (BO) of 10 items [52]. As there is no composite score for the entire scale, this method allows each subscale to be used independently. Based on previous research [46], some wording was modified to suit the study on both people and animals, for example, “I like my work as [carer]/helper”. Each item used a 5-point Likert scale ranging from “1 = never” to “5 = very often” to reflect the frequency of the experiences in the last 30 days. The Cronbach’s alpha was recorded at 0.74 for the whole measure, and CS at 0.87, BO at 0.72 and CF at 0.80.

Resilience. Resilience (RS) was measured by the Brief Resilience Scale (BRS) [58]. This scale specifically measures “the ability to bound back” or recover from stress. It consists of six questions with an equal number of positively and negatively worded questions, using a 5-point Likert scale from “1 = strongly disagree” to “5 = strongly agree”. Cronbach’s alpha was reported at 0.88.

Satisfaction as an oil spill responder. Role satisfaction (RSat) was measured by three items derived from Brief Job Satisfaction Measure II by Judge et al. [59] and the Job Satisfaction Scale [60] was adapted to measure participants’ satisfaction as an oil spill responder. A 5-point Likert scale was used to measure the level of agreement, ranging from “1 = strongly disagree” to “5 = strongly agree”. Cronbach’s alpha was reported at 0.95.

Life satisfaction. Life satisfaction (LS) was measured by using two questions from Quinn and Staines [61]. The questions were “taking all things together, how happy would you say you are with your life?” and “in general, how satisfying do you find the ways you’re spending your life these days?” Response options were from “1 = not too happy” to “3 = very happy”. Cronbach’s alpha was reported at 0.83.

Socio-demographics. Participants reported their age group, gender, education, occupation and income. Two questions were adapted based on Kidd et al. [62] and Yeung et al. [46] to ask when their interest arose in and what age they began volunteering and caring for injured animals and/or wildlife.

Open-ended comments. Free text boxes were provided to ask participants to provide written comments on “what information/skills would you have liked to receive in pre-training to prepare you better for your work in the spill?”, “responses on whether they felt overscheduled at any point”, and “any equipment, facilities, process or procedure that may have affected the job”.

Due to the exploratory nature of the questionnaire, no prior power testing was conducted. Data were exported from SurveyMonkey (San Mateo, CA, USA) into Microsoft Excel (Microsoft Corporation, Redmond, WA, USA) spreadsheets and recoded as necessary. The IBM SPSS Statistical package (version 25, IBM SPSS Statistics for Windows, IBM, Armonk, NY, USA) was used for data entry and analysis. For each subscale of the ProQOL, raw scores were summed (after several items were reverse-scored) and converted into *t*-scores in which each scale had a mean of 50 and a standard deviation of 10 [52]. In addition, each scale’s *t* score was recoded into a dichotomous variable to report the level of severity for CS, BO and CF. Stamm’s [52] recommendations were followed to define “high risk”, a *t* score below 43 for the lowest quartile for CS, medium risk or protective represented in the range of 43 and 56, and *t* scores at 57 or above for the highest quartile for BO and CF.

All other variables in the study were assessed using descriptive analyses including comparisons of mean, standard deviation, percentage, and range. Chi-square was used to assess if there were any significant differences among socio-demographic variables between oil spills groups. While this was a pilot study, there were only a small pool of experts in the area of oil spill wildlife rescue. Hence, some of the independent variables were further dichotomised into the following categories: age (≤49; ≥50), highest education achieved (post-secondary or less; Bachelor’s degree or higher), oil spill groups (NZ Rena; US Refugio); and responder role (staff; volunteers). Bivariate correlations were performed to explore relationships between variables using Spearman’s correlation coefficients. Mann–Whitney U tests were employed to test for differences in Likert-based responses between two independent groups. Responses to the free-text question were reported to complement the quantitative data.

## 3. Results

Most of the responders reported to be aged 40 and over, and female. The responders were highly educated with over 60% having university degree. Half of the NZ responders worked with/for animals as part of their main job but over 60% of the US responders’ main jobs were involved with other tasks such as emergency management, environmental consulting or retired. Eighty percent of all respondents reported to have enough and more than enough total income to meet their everyday needs. Over 60% of the respondents were employed for wages. Many of the NZ responders reported to have become interested in caring for animals/wildlife since early childhood (61.8%) while it was since adolescence for the US responders (56.3%). NZ respondents seemed to have started volunteering to care for injured animals or wildlife under the age of 29 (64.5%) whereas US respondents reported starting at a later age (>40). No significant difference was detected in any of socio-demographic variables between NZ and US oil spill groups. Table 1 shows detailed demographic information about the background of the responders.

A majority of the responders had attended a small number of oil spills (e.g., five or less). Over 40% of NZ responders received notice regarding oil spills from the National Oil Wildlife Response Team, Maritime NZ or Wildbase, Massey University followed by other avenues such as NZ Department of Conservation, other government agency and NZ regional councils; US responders received their notice mainly through OWCN or their member organisation. Most of NZ responders responded in the oil spill as paid staff (73.5%) while over 80% the US responders acted as volunteers (paid or unpaid). More than 70% of all respondents reported to have been given clear indication of their positions to fill during the spill and had an accurate vision of tasks and responsibilities. More than half of the responders reported that their main role was in wildlife rehabilitation facility for primary care. Moreover, US responders involved more with wildlife field operations, reconnaissance, recovery or hazing (50%) and wildlife transportation (12.5%) than NZ responders in these two areas (23.5%; 8.8%). Over 50% of NZ responders participated in-person workshop on oiled wildlife training prior to the spill while most US responders (>75%) reported to have participated in either or both online and/or in-personal training. Just over 70% of NZ responders supervised other staff or volunteers as part of the response compare with only 50% of US responders. Both cohorts reported feeling they had enough information and direction “most of the time” to “all the time” (>70%) to adequately guide those they were responsibility for. Table 2 illustrate the description on respondents’ involvement with previous oil spill incidents.

When asked what information/skills they would have liked to receive in pre-training to prepare them to work better in the spill, most of NZ responders commented on areas relating to human resources, including staff, and rosters. Another area that received comments from NZ responders was roles and responsibilities, including Coordinated Incident Management System (CIMS) and communication skills, in which respondents stated “a clearer picture of people’s positions and associated responsibilities” and “the onsite hands-on experience with guidance from experienced responders worked well”. When commenting on working environment, both NZ and US respondents simultaneously reported the importance of physical fitness to endure the oil spill incidents, “basic and advanced fitness: standards for physical expectations for various positions (NZ)” and “I did not expect the degree of heat & humidity within the facility, nor how wearing PPE would add to that, resulting in near syncope (US)”. In addition, two specific comments on stress and emotion management came from US responders stating “the overall training helps to prepare for the spills. I think what happens when you return to the ‘normal’ life training needs to include compassion stress information and techniques on how to get back into a regular routine” and “perhaps an understanding of how working as a close team in an emergency situation can impede relations at home when loved ones are not directly involved in the situation and the spill workers are tired”.

NZ responders reported they worked consecutively between five and 10 days during the spills (47.1%) while US responders worked up to five days (50%). Most of the responders reported to have adequate breaks when working the spill (>70%). Over 60% of NZ responders found the workload during the oil spill incident to be high when compared with 37.5% from US responders. Both cohort of responders reported feeling their skills were used effectively and suited to the tasks assigned “most of the time” to “all the time” (>80%). Although less than 13% of the responders felt overscheduled at any point, some participants commented that high workload was involved during the initial phase of response but once routines were established and people knew what their roles were, it was easy to follow. Meanwhile, at least three NZ responders mentioned they felt overwhelmed rather than overscheduled due to poor communication and too many people with too many requests in the beginning stage. Some of the comments derived from both US and NZ responders who were tasked with management roles reported that they did not feel overscheduled but at times it was hard to manage people who were required to attend in multiple meetings at the same time and to get adequate cover/replacement for their areas.

More than 50% of the responders reported that they received regular updates about the entire spill process and progression. Over 85% reported to have received regular updates about their work area. All US responders felt they received regular updates about how they fitted into the bigger picture compared with only 73.6% for NZ responders. More than 80% reported receiving regular reminders of safety policies and potential hazards. All US responders indicated they have found the experience responding to the oil spill to be rewarding when compared with 88.2% from NZ responders. One responder wrote “it was something I had never experienced before, the satisfaction of caring for wildlife was immense”. Over 90% of responders said they would participate in oil spill response again in the future. Table 3 shows respondents’ view on workload, support and overall experiences in helping during the oil spills.

When asked what made the job easier or harder, 22 comments were related to commending the effectiveness of the workflow to make the operation smooth and easy to deal with. Most of the comments by both groups focused on procedures, tasks, planning and teamwork, for example “processes and procedures developed for Coordinated Incident Management Systems”, “good support crew and secondary managers”, “familiar with team members, following normal Incident Management Team (IMT) planning cycle”, “the full-time staff, their support, direction & supervision were integral for safe practice and protocol compliance” and “the ease with which we could acquire the things we needed (e.g., PPE, stationery, etc.), which freed up additional time to care for animals”.

Meanwhile, communication was identified as both a facilitator and barrier in processes when dealing with animal recovery, cleaning, and other response functions. The importance of good communication and working with good people were emphasized strongly by both groups, such as “excellent guidance by staff during the cleaning of the oiled birds”, “working with a committed contractor who was prepared to go the extra mile to help”, and “presence of experienced responders from abroad, positive and supportive colleagues to optimize the environment of the recovering birds”. On the other hand, lack of or poor communication and management of people and processes were reported to make the job challenging during oil spill incidents, with comments including “communication was very poor between field ops and the clinic”, “not knowing or having access to procedure…poor supply of information and logistic support to acquire equipment in a timely fashion”, and “the lack of species-specific supplies and protocols made it challenging to provide effective care initially”.

Having essential facilities for comfort, safety, rest, and food were also highly valued to help with their working environment during the oil spills incidents. One responder admitted to having “little appreciation for degree of difficulty working in oil spill”, but both groups shared similar comments that having things such as “protective gear”, “lots of food and beverages”, “having decent accommodation and food to have a good night sleep” helped making easier for the job and to cope with the situation. However, there were also some comments on people not getting enough rest, lack of specific equipment for transportation and to avoid contamination, and lack of electronic system to enter information, which put more pressure on the jobs, such as “accommodation was changed regularly which meant you couldn’t fully unpack or relax”, “lack of access to equipment such as printers”. “We were using a paper-based system that was slow and cumbersome”, and “sometimes there were too many dead animals to recover and document. The cetaceans were too heavy for 4 people to walk with”.

When examining the perceived effect from the number of participants experiencing worsened or improved life conditions in response to helping in the oil spills, NZ responders scored 20.6% (*n* = 7) in worsened physical health, 8.8% (*n* = 3) worsened social relationships with others and 8.8% (*n* = 3) worsened financial situation when compared with US responders as 0% (*n* = 0), 6.3% (*n* = 1) and 0% (*n* = 0), respectively. In terms of ProQOL, the cut scores are set at 25th, 50th, and 70th percentile to indicate relative risks or protective factors (Stamm, 2010). Of note, no responder scored in the high-risk range for BO, while 30% (*n* = 15) scored in the medium risk, and 66% (*n* = 33) were at low risk. Similar to BO, no responder scored in the high-risk range for CF, while 26% (*n* = 13) scored in the medium range, and 70% (*n* = 35) were in the low risk range. For CS, 50% (*n* = 25) of the responders scored high and 46% (*n* = 23) were in the moderate range and none in the low level. Overall, responders indicated a normal RS status (M = 3.74; SD = 0.70). Responders scored very high in their RSat as oil spill responders (90 to 92%) and LS (96 to 100%). Table 4 shows descriptive statistics for ProQOL, RS, RSat and LS.

RS was significantly and positively correlated with CS (*r* = 0.36), RSat (*r* = 0.38) and LS (0.32) but negatively with BO (*r* = −0.51), and CF (*r* = −0.44). CS was significantly and negatively correlated with BO (*r* = −0.74) but not with CF; it was also negatively correlated with gender (*r* = −0.31) but positively with RSat (*r* = 0.45), LS (*r* = 0.41), age group (*r* = 0.29), responder group (*r* = 0.32) and role (*r* = 0.40). BO was significantly and positively correlated with CF (*r* = 0.59) but negatively with resilience (*r* = −0.41), age group (*r* = −0.35), and responder group (*r* = −0.39). CF was only found to be significantly and negatively correlated with responder group (*r* = 0.37). Table 5 shows a correlation matrix.

The Mann–Whitney *U* tests were used to compare ProQOL’s three subscales (BO, CS and CF), perceived effect of helping in oil spill, RS, RSat and LS for gender, age groups (≤49; ≥50), oil spill groups (NZ Rena; US Refugio) and responder role (staff; volunteers). In terms of gender differences, results showed three significant differences in CS, RSat and LS. Female respondents experienced higher level of CS than male respondents, and the effect size was medium. Females respondents showed higher levels in both RS and LS than males, which also indicated medium effect size. Age group differences were only found in the older age group (≥50), scoring higher in RSat and lower in BO than those who are 49 years of age or younger, reporting medium effect size.

When comparing the variables between the oil spill groups, US responders reported to score higher in RSat, LS, and CS than NZ responders. In contrast, NZ responders scored higher in BO and CF. The effect sizes were all recorded at medium.

Two significant differences were detected in perceived effect in helping oil spills and CS among responder role. Participants who responded as staff reported to have higher perceived effect from the oil spill incidents than volunteers. Volunteers reported to have higher level of CS than staff. Both effect sizes were medium. No significant differences were detected in the variables among education levels. Table 6 shows summary of differences on Mann–Whitney Test.

## 4. Discussion

Numerous oil spills have affected global coastal communities and their wildlife due to human-induced accidents [28]. Oiled wildlife response in some areas of the world is considered an ethical responsibility to minimize suffering to wildlife and the role of oiled wildlife responders is critical for wildlife recovery, rehabilitation, and survival [29]. Despite the crucial role oiled wildlife responders have played in oil spill incidents, to our knowledge, this is the first study that examined their characteristics and experiences as they relate to mental health and professional quality of life. In this retrospective study, the aims were to explore the perspectives of oiled wildlife responders who participated in the NZ MV Rena and US Refugio oil spills, including the importance of their health and wellbeing in responding to disaster, and to suggest strategies that can help in their roles to rescue, recover, rehabilitate wildlife in the future.

Results of the study showed that the profile of the oiled wildlife responders between NZ and US responders was quite similar, with participants from both countries being mainly older (aged >40 and over), female, highly educated, and with good financial capacity due to waged employment. The main task responders were involved in during the spills was primary care with wildlife. Most responders attended one to five oil spill incidents but reported similar and positive views regarding operations during a spill response, e.g., enough information, directions and role clarification, adequate breaks, their skills being used effectively, received regular updates, found the experience rewarding, and keen to participate in oil spill response again.

No responders from either cohort indicated any severe impacts in the level of their health and wellbeing such as BO and CF. However, the US responders overall reported less effects in relation to their mental health, physical health, social relationships, and financial situation when compared with the NZ responders. In addition, the US responders scored significantly better in CS, RSat and LS than the NZ responders. A reason for this maybe that over 80% of the US responders identified themselves as volunteers in responding to the oil spills. Volunteers were found to have significantly higher level of CS compared to staff across both spills. Previous research has found that people who volunteered in disaster relief work reported positive mental health responses as a result of helping those in need [63,64]. Volunteers who mostly comprised in the US responders in the current study may be less susceptible to burnout and compassion fatigue due to the job motivation and satisfaction coming from volunteering [65] as the participation in oil spill incidents is not a regular occurrence. Carrying on a volunteering activity for a long time, however, can trigger high levels of emotional exhaustion and this can often be the first step in the process leading to burnout syndrome [66] so this should be taken into consideration and monitored in long term volunteers.

Our results regarding the relationships between the ProQOL variables (CF, BO and CS) and RS supported previous research. We found that RS was highly correlated with CF, BO and CS, consistent with existing research [54,67]. Our study showed that responders who possessed the capacity to adapt to and maintain balanced psychological and emotional status, felt satisfied in the role/work and life in general tended to be less susceptible to the negative effects derived from responding to disaster events. Although higher BO was associated with higher CF, the lower BO scores in the current study could be related to participants viewing that responding to disaster, such as oil spill incidents, is simply part of their job. Especially given they have received training and have the tools to provide further resources or referrals. Furthermore, the insignificant association between CF and CS could signal that the oiled wildlife responders may be more at risk for burnout because of working overload and overtime while CF relates to susceptibility to others’ trauma experience [68]. Therefore, it makes sense to avoid the negative effects of compassion fatigue by improving compassion satisfaction and addressing burnout. Some of the recommendations have been made about how to decrease BO, such as lessening workload and avoiding extended working hours, providing psycho-social interventions, debriefing through formalized groups and ensuring professional capacity training to be implemented [49,69,70]. When support opportunities are available, they can help people to develop experience, understanding and resilience to enhance compassion satisfaction.

Not surprisingly, age was found to have significant relationships with CS and BO, further confirming that older responders tend to have more life experiences and the maturity are likely to enhance their ability to handle difficult circumstances [46]. Interestingly, female responders were found to have higher CS than male. More women are engaging in disaster management and rescue operations, and research has shown their resilience in leadership and grassroots organizing [71,72] are becoming more prevalent to bring long-term changes. Women have been shown to build more participation spaces and influence decision-making processes [73], and these experiences may have contributed more from the work engagement to enhance CS. On another note, there was no significant difference between males and females on CF and BO, which aligned with some of the existing research [49,55]. However, this result was also in contrast to much of the research regarding gender differences for these variables [74,75,76]. Two possible explanations for this lack of gender difference may be due to firstly, the profile of the current cohort in which responders have attended training workshops prior to the deployment of oil spill incidents. They may have also received other training in crisis management incidents or crisis intervention methods for other disaster events in the past. Atkins and Burnett [77] found that training may have the potential to build resilience and act as a protective factor for CF or BO among disaster respondents. Research have indicated that this type of training may help reducing gender differences among this population [78,79]. Secondly, it may be due to an inherent selection bias in the survey set, as oiled wildlife responders are not reflective of the public as a whole. Most people going into this field, regardless of gender, have high empathy and are committed in this kind of work, so gender predilections may be already removed.

Individual responders’ capacities are important to improve health and wellbeing, but so too are the organizational and societal context in which responses operate in during an oil spill incident. Qualitative responses support the quantitative analysis, indicating that when response initiation and operations, communication, support from co-workers or teams, and infrastructure were well-prepared and coordinated, these factors could contribute to enhanced CS and RSat. Participants’ comments on situation awareness, leadership and teamwork, expert knowledge of emergency response procedures and management style were also reflected in research on how to enhance coordinating knowledge in crisis situations [41]. Previous research has stated that perceived organization support, social support and better relationships with co-workers are associated with lower levels of CF and higher level of RS [80,81]. Oiled wildlife responders are a unique group of professionals with specialized skills and knowledge and it is in an organization’s or government’s best interest to increase the professional quality of life of these experts to promote successful responses and sustained volunteer involvement. This study has shown that individuals with low level of RS may struggle to adapt to the stressful and exhausting demands of the disaster response field. Hence, maintaining an optimal level of RS is important for critical incident responders to buffer the adverse effects of CF and BO. A factor that must be taken into consideration for this research is that the responders that answered our questionnaire are individuals who operated during a response under the only two full-time, government integrated and university-contracted oiled wildlife response teams in the world. The OWCN managed by the University, California at Davis, and Wildbase, coordinated by Massey University, both have full time staff who coordinate and train volunteers and staff, and who have oiled wildlife response plans, strategies, processes and equipment for their country or state. This means the responses being asked about in this research were likely to have had better preparedness and coordination than many other responses that do not have full time oiled wildlife response emergency response teams in their region. The preparedness advantages of working under these two organizations would have included less average response time for wildlife, therefore likely higher survival of wildlife in the response and this could have had an effect on the variables explored in the current study. Organizations that regularly receive individual oiled animals from natural seps or minor incidences (therefore ongoing training and knowledge how to best help species), rescue and rehabilitation wildlife at pre-established facilities or identified locations for mobile facilities (therefore the challenges of at-the-time constructing/modifying of rehabilitation facilities were not present), and, for both of these incidences, that the total number of animals at the facilities were within the capabilities of the facilities, i.e., not overwhelming, would have helped and prepared responders to deal with these situations. Additional to these advantages of working or volunteering for prepared response organizations, these two spills were single source, slow moving spills involving wildlife that are known to have high rates of success for rehabilitation and survival. Responses would likely be very different if participants had had to respond to incidents where lack of any infrastructure led to euthanasia of large numbers of wildlife or where tens of thousands of animals were being cared for without adequate preparedness or facilities [29].

There is increasing evidence that oil spills are recognized as significant predictors to environmental degradation and wildlife mortality, emphasizing that planning, preparedness and prevention are keys to minimizing these impacts [28,29]. Recent research has revealed that wildlife response and rehabilitation during oil spills is beneficial both to individual animals as well as entire species in certain situations, as post-release evidence shows survival and reproductive rates at the same levels as never oiled individuals with positive impacts among rehabilitated wildlife after an oil spill [82,83,84]. These studies show the importance of how oiled wildlife responders can make significant contribution to uphold the ethical and legal commitment to care for wildlife and their intrinsic values. As such, evidence in this study strongly suggests that it is important that government, organizations and industry build a resilient oiled wildlife responder workforce even before crisis happens to ensure that they would be able to cope if an event were to happen.

There are several limitations to this study, firstly being the relatively small sample size. The current study relied on a purposive and convenience sample of oiled wildlife responders that attended the NZ MV Rena or US Refugio oil spills. Convenience sampling and small sample sizes can potentially raise questions about potential biases relating to the representativeness of the respondents. Furthermore, our research was limited by self-reported survey data based on retrospective experiences and their re-collection of their experiences may have been biased or incomplete. There were some anecdotes that during the NZ MV Rena oil spills, some of the oiled wildlife responders expressed stress and frustration; hence, the interest of developing the current research. Given that MV Rena and US Refugio happened almost 10 and six years ago, respectively, some of the responders may have sought other support and professional development to enhance their current status and coping mechanisms. Due to the time lag, it was impossible to conduct cross-correlational analysis where negative impacts could be revealed. Future research should implement pre-and post-emergency response surveys and use longitudinal methodology to track the progress and development of the responders in order to develop more responsive support for those who wish to work as oiled wildlife responders. What is clearly stated in this study is where the participants were positively supported but further information is needed in where this was not the case. Although some qualitative comments were collected, they were purely illustrative to complement the quantitative data. Qualitative approach is recommended to explore the lived experience of oiled wildlife responders in future research.

## 5. Conclusions

Past research shows that oiled wildlife response needs to focus on planning and preparedness [85]. The current research provides additional findings that indicate the importance of appropriate pre-spill training promotion of good communication and supportive organisational relationships as part of ongoing professional development to equip staff and regular volunteers with knowledge, skills and coping strategies [18,41]. The field of disaster response, such as oil spill incidents, places high demands on responders working within high-stress and unpredictable environments. They may experience adverse and harmful effects of direct or indirect trauma such as CF and BO, which can erode their ability and resilience to fulfil their role and subsequently impact on their own personal, physical, and psychosocial health. Working within such environment will always be stressful for oiled wildlife responders. This study shows, however, that oiled wildlife responders can benefit from professional training, supported by organizations and wider networks, in the skills needed for performance when under stress [86], early recognition of danger and error-recovery [87], self-care, and capacity building [88].

## Figures and Tables

**Table 1:** Oil spill respondents background and demographic information.

Variables	N (%)	
	NZ	US
What is your current age?		
20–29	1 (2.9)	0 (0.0)
30–39	5 (14.7)	1 (6.3)
40–49	9 (26.5))	3 (18.8)
50–59	13 (38.2)	4 (25.0)
60–69	3 (8.8)	4 (25.0)
70+	3 (8.8)	4 (25.0)
Gender		
Female	19 (55.9)	13 (81.3)
Male	15 (44.1)	3 (16.7)
Highest education qualification?		
Secondary school qualifications	3 (8.8)	0 (0.0)
Post-secondary qualifications	9 (26.5)	1 (6.3)
University degree	21 (61.8)	12 (75.0)
Other	1 (2.9)	3 (18.8)
Do you work with animals or in oil spill as part of your main job?		
Work with/for animals	17 (50.0)	7 (43.8)
Oil spill response	5 (14.7)	4 (25.0)
Other	10 (29.4)	10 (62.5)
How total income meet everyday needs?		
Not enough	3 (8.8)	2 (12.5)
Just enough	4 (11.8)	1 (6.3)
Enough	20 (74.1)	7 (43.8)
More than enough	7 (20.6)	6 (37.5)
Your current employment		
Volunteer work	2 (5.9)	1 (6.3)
Employed for wages	25 (73.5)	10 (62.5)
Other	7 (20.6)	5 (31.3)
When have you become interested in caring for animals/wildlife?		
Since early childhood	21 (61.8)	5 (31.3)
Since adolescence	12 (35.3)	9 (56.3)
Since adulthood	0 (0.0)	0 (0.0)
Other	1 (2.9)	2 (12.5)
Age to volunteer and care for injured animals or wildlife?		
<29	20 (64.5)	7 (43.8)
30–39	3 (9.7)	0 (0.0)
40–49	5 (16.1)	4 (25.0)
50–59	3 (9.7)	2 (12.5)
60–69	0 (0.0)	3 (18.8)

**Table 2:** Involvement with previous oil spill incidents.

Variables	N (%)	
	NZ	US
How many oil spills attended?		
1–5	29 (85.3)	16 (100.0)
5–10	1 (2.9)	0 (0.0)
>10	4 (11.8)	0 (0.0)
Who initially activated you to the spill?		
Through OWCN or member organisation	1 (2.9)	14 (87.5)
NOWRT/Wildbase, Massey University	15 (44.1)	0 (0.0)
Maritime NZ	9 (26.5)	0 (0.0)
US Government agency	0 (0.0)	0 (0.0)
Other	11 (32.4)	3 (18.8)
Did you respond as staff or volunteer?		
Staff (paid)	25 (73.5)	6 (37.5)
Volunteer (paid)	6 (17.6)	2 (12.5)
Volunteer (unpaid)	2 (5.9)	8 (50.0)
Volunteer (expenses only)	1 (2.9)	3 (18.8)
Other	0 (0.0)	1 (6.3)
Were you given a clear indication of your position to fill?		
Yes	24 (70.6)	12 (75.0)
No	8 (23.5)	3 (18.8)
Other	2 (5.9)	1 (6.3)
Which part of the oil response did you get involved?		
Wildlife field operations, reconnaissance, recovery or hazing	8 (23.5)	8 (50.0)
Wildlife field stabilisation	8 (23.5)	4 (25.0)
Wildlife rehabilitation facility—primary care	18 (52.9)	12 (75.0)
Wildlife transportation	3 (8.8)	2 (12.5)
Planning	3 (8.8)	0 (0.0)
Logistics	2 (5.9)	0 (0.0)
Non-Wildlife operations	3 (8.8)	0 (0.0)
Other	15 (44.1)	0 (0.0)
Did you have an accurate vision of what your tasks and responsibilities?		
Yes	26 (76.5)	14 (87.5)
No	4 (11.8)	2 (12.5)
Other	4 (11.8)	0 (0.0)
Did you participate in any oiled wildlife trainings prior to the spill? *		
Online or webinar training	4 (11.8)	12 (75.0)
In-person workshops	18 (52.9)	14 (87.5)
Other relevant training	11 (32.4)	3 (18.8)
Did you supervise other staff or volunteers as part of the response?		
Yes	24 (70.6)	8 (50.0)
No	10 (29.4)	8 (50.0)
Did you feel you had enough information and direction to adequately guide those you were responsible for?		
Not at all	1 (2.9)	0 (0.0)
Sometimes	5 (14.7)	1 (6.3)
Most of the time	18 (52.9)	7 (43.8)
All the time	7 (20.6)	6 (37.5)

* Multiple answers allowed.

**Table 3:** Workload, support and overall experiences.

Variables	N (%)	
	NZ	US
How many consecutive days did you work during the spill?		
0–5	3 (8.8)	8 (50.0)
5–10	16 (47.1)	4 (25.0)
10–15	9 (26.5)	3 (18.8)
>16	6 (17.6)	1 (6.3)
Did you have adequate breaks on the days that you were working the spill?		
Not frequent enough	7 (20.6)	0 (0.0)
Adequate	25 (73.5)	16 (100.0)
Too frequent	1 (2.9)	0 (0.0)
How did you find the workload during the oil spill incident?		
Low	2 (5.9)	2 (12.5)
Medium	10 (29.4)	8 (50.0)
High	22 (64.7)	6 (37.5)
Did you feel your skills were used effectively and suited to the tasks assigned?		
All the time	11 (32.4)	7 (43.8)
Most of the time	16 (47.1)	8 (50.0)
Sometime	7 (20.6)	1 (6.3)
Not at all	0 (0.0)	0 (0.0)
Did you feel overscheduled at any point?		
Not at all	15 (44.1)	10 (62.5)
Sometimes	15 (44.1)	4 (25.0)
Most of the time	3 (8.8)	2 (12.5)
All the time	1 (2.9)	0 (0.0)
Receive regular updates about the entire spill process and progression?		
Not at all	1 (2.9)	1 (6.3)
Sometimes	9 (26.5)	7 (43.8)
Most of the time	13 (38.2)	6 (37.5)
All the time	11 (32.4)	2 (12.5)
Receive regular updates about your work area?		
Not at all	0 (0.0)	0 (0.0)
Sometimes	3 (8.8)	2 (12.5)
Most of the time	13 (38.2)	9 (56.3)
All the time	18 (52.9)	5 (31.3)
Receive regular updates about how you fitted into the bigger picture?		
Not at all	3 (8.8)	0 (0.0)
Sometimes	6 (17.6)	0 (0.0)
Most of the time	9 (26.5)	10 (62.5)
All the time	16 (47.1)	6 (37.5)
Receive regular updates about reminders of safety policies and potential hazards?		
Not at all	1 (2.9)	0 (0.0)
Sometimes	4 (11.8)	1 (6.3)
Most of the time	11 (32.4)	5 (31.3)
All the time	18 (52.9)	10 (62.5)
Overall, how much of a rewarding experience did you find responding to the oil spill?		
Not at all	1 (2.9)	0 (0.0)
To some extent	3 (8.8)	0 (0.0)
Mostly	5 (14.7)	2 (12.5)
Definitely	25 (73.5)	14 (87.5)
Would you participate in oil spill response again in the future?		
Yes, definitely	28 (82.4)	15 (93.8)
Maybe	5 (14.7)	0 (0.0)
Not sure	0 (0.0)	0 (0.0)
Not at all	1 (2.9)	1 (6.3)

**Table 4:** Descriptive statistics for ProQOL, resilience, role satisfaction and life satisfaction for all participants.

Variables	Range	Mean	SD
ProQOL (Compassion Fatigue Subscales)			
Compassion Satisfaction (CS)	31.61–67.47	41.85 (*t* = 57)	4.82
Burnout (BO)	30.38–76.47	20.52 (*t* = 43)	5.53
Compassion Fatigue (CF)	31.56–78.29	18.94 (*t* = 43)	5.05
Resilience—6 items	2.50–5.00	3.74	0.70
	% (agree to strongly agree)		
Satisfaction being an oil spill responder			
Most days I am enthusiastic about my job/role as an oil spill responder	92	4.47	0.68
I find real enjoyment in my job/role as an oil spill responder	90	4.52	0.68
Taking everything into consideration, I am satisfied with my job/role as an oil spill responder	92	4.46	0.71
Life satisfaction			
Taking all things together, how happy would you say you are with your life?	100	2.60	0.50
In general, how satisfying do you find the ways you are spending life these days?	96	2.52	0.58

**Table 5:** Relationships between demographic and group characteristics with resilience, compassion satisfaction, burnout and compassion fatigue.

Variable	RS	CS	BO	CF	RSat	LS	Age	Gender	Education	NZ/ US	S/V
	*r*	*r*	*r*	*r*	*r*	*r*	*r*	*r*	*r*	*r*	*r*
RS	--	0.36 *	−0.51 **	−0.44 **	0.38 **	0.32 *	0.10	−0.03	−0.19	0.10	0.21
CS		--	−0.74 **	−0.12	0.45 **	0.41 **	0.29 *	−0.31 *	−0.19	0.32 *	0.40 **
BO			--	0.59 **	−0.41 **	−0.56 **	−0.35 *	0.22	0.22	−0.39 **	−0.28
CF				--	−0.25	−0.27	−0.22	−0.03	0.17	−0.37 **	−0.09

Bivariate correlation—Abbreviations: RS—resilience, CS—compassion satisfaction, BO—burnout, CF—compassion fatigue, RSat—role satisfaction, LS—Life satisfaction, S/V—staff or volunteer. Age (1 = ≤49; 2 = ≥50); Gender (1 = Female; 2 = Male); Education (1 = post-secondary or less; Bachelor’s degree or higher); S/V (1 = staff; 2 = volunteer). * *p* < 0.05 (2-tailed). ** *p* < 0.01 (2-tailed); *r* = 0.1–0.29 small; *r* = 0.3 medium; *r* = 0.5 large correlation.

**Table 6:** Summary of differences among variables on Mann–Whitney Test.

Variables	Median	Median			
	Male (*n* = 17)	Female (*n* = 31)	Z-Value	*p*-Value	*r*
Compassion Satisfaction (CS)	39.00	42.00	−2.21	0.03	0.31
Resilience (RS)	4.00	5.00	−2.57	0.01	0.37
Life Satisfaction (LS)	2.00	3.00	−2.20	0.03	0.31
	Age ≤ 49 (*n* = 30)	Age ≥ 50 (*n* = 19)			
Role Satisfaction (RSat)	4.00	5.00	2.58	0.01	0.37
Burnout (BO)	22.00	18.00	−2.37	0.02	0.34
	US (*n* = 15)	NZ (*n* = 34)			
Role Satisfaction (RSat)	5.00	4.17	2.60	0.01	0.37
Life Satisfaction (LS)	3.00	2.50	2.06	0.04	0.29
Compassion Satisfaction (CS)	44.50	40.00	2.19	0.03	0.32
Burnout (BO)	17.00	22.00	−2.70	0.01	0.39
Compassion Fatigue (CF)	16.00	17.00	−2.25	0.02	0.33
	Staff (*n* = 31)	Volunteer (*n* = 17)			
Perceived effect	2.93	2.71	−3.21	0.00	0.45
Compassion Satisfaction (CS)	40.00	45.00	2.76	0.01	0.40

## Data Availability

These data are not publicly available.

## References

[1] Li P., Cai W., Lin W., Chen B., Zhang B. (2016). Offshore oil spill response practices and emerging challenges. Mar. Pollut. Bull..

[2] Drescher C.F., Schulenberg S.E., Smith C.V. (2014). The Deepwater Horizon oil spill and the Mississippi Gulf Coast: Mental health in the context of a technological disaster. Am. J. Orthopsychiatry.

[3] Murphy C., Doty I., Moore A. An Overview of Volunteer Efforts During the Refugio Oil Spill Incident in Santa Barbara, California. Proceedings of the International Oil Spill Conference Proceedings.

[4] Lowe S.R., Bonumwezi J.L., Valdespino-Hayden Z., Galea S. (2019). Posttraumatic stress and depression in the aftermath of environmental disasters: A review of quantitative studies published in 2018. Curr. Environ. Health Rep..

[5] D’Andrea M.A., Reddy G.K. (2018). The development of long-term adverse health effects in oil spill cleanup workers of the Deepwater Horizon Offshore drilling rig disaster. Front. Public Health.

[6] Aguilera F., Mendez J., Pasaro E., Laffon B. (2010). Review on the effects of exposure to spilled oils on human health. J. Appl. Toxicol..

[7] GuLF STUDY The GuLF STUDY—A Long Term Health Study for Oil Spill Clean-Up Workers and Volunteers. https://gulfstudy.nih.gov/en/index.html.

[8] D’Andrea M.A., Reddy G.K. (2014). Health risks associated with crude oil spill exposure. Am. J. Med..

[9] Laffon B., Pasaro E., Valdiglesias V. (2016). Effects of exposure to oil spills on human health: Updated review. J. Toxicol. Environ. Health Sci. Part B.

[10] Rung A.L., Gaston S., Robinson W.T., Trapido E.J., Peters E.S. (2017). Untangling the disaster-depression knot: The role of social ties after Deepwater Horizon. Soc. Sci. Med..

[11] Stroope S., Slack T., Kroeger R.A., Sweet Keating K., Beedasy J., Sury J., Brooks J., Chandler T. (2021). Deepwater Horizon oil spill exposure, industry sector, and child health. Pop. Res. Pol. Rev..

[12] Choi K.H., Lim M.H., Ha M., Sohn J.N., Kang J., Choi Y., Cheong H. (2016). Psychological vulnerability of residents of communities affected by the Hebei spirit oil spill. Disaster Med. Public Health Prep..

[13] Rung A.L., Oral E., Fontham E., Harrington D.J., Trapido E.J., Peters E.S. (2015). Mental health impact of the Deepwater Horizon oil spill among wives of clean-up workers. Epidemiology.

[14] Genuis S.J. (2012). What’s out there making us sick?. J. Environ. Public Health.

[15] Buttke D., Vagi S., Bayleyegn T., Sircar T., Morrison M., Wolkin A. (2012). Mental health needs assessment after the Gulf Coast oil spill—Alabama and Mississippi. Prehospital Disaster Med..

[16] Osofsky H.J., Osofsky J.D., Hansel T.C. (2011). Deepwater horizon oil spill: Mental health effects on residents in heavily affected areas. Disaster Med. Public Health Prep..

[17] Moore R., Burns C. (2011). The effects of oil spills on workers involved in containment and abatement: The role of occupational health nurse. AAOHN J..

[18] Brooks S.K., Dunn R., Amlot R., Greenberg N., Rubin G.J. (2016). Social and occupational factors associated with psychological distress and disorder among disaster responders: A systematic review. BMC Psy..

[19] D’Andrea M.A., Reddy G.K. (2013). Health consequences among subjects involved in Gulf Oil spill clean-up activities. Am. J. Med..

[20] D’Andrea M.A., Reddy G.K. (2014). Crude oil spill exposure and human health risks. J. Occup. Environ. Med..

[21] Jacklitsch B.L., King K.A., Vidourek R.A., Merianos A.L. (2019). Heat-related knowledge, perceptions and barriers among oil spill cleanup. Saf. Sci..

[22] Kwok R.K., McGrath J.A., Lowe S.R., Engel L.S., Jackson W.B., Curry M.D., Payne J., Galea S., Sandler D.P. (2017). Mental health indicators associated with oil spill response and clean-up: Cross-sectional analysis of the GuLF STUDY cohort. Lancet Public Health.

[23] Palinkas L.A. (2012). A conceptual framework for understanding the mental health impacts of oil spills: Lessons from the Exxon Valdez oil spill. Psychiatry.

[24] Palinkas L.A., Petterson J.S., Russell J., Downs M.A. (1993). Community patterns of psychiatric disorders after the Exxon Valdez oil spill. Am. J. Psychiatry.

[25] Brooks S.K., Dunn R., Sage C.A.M., Amlot R., Greenberg N., Rubin J. (2015). Risk and resilience factors affecting the psychological wellbeing of individuals deployed in humanitarian relief roles after a disaster. J. Ment. Health.

[26] James K. (2011). Introduction to the special issue: Terrorism, disaster, and organizational science. J. Organ. Behav..

[27] Benedek D.M., Fullerton C., Ursano R.J. (2007). First responders: Mental health consequences of natural and human-made disasters for public health and public safety workers. Annu. Rev. Public Health.

[28] Chilvers B.L., Morgan K.J., White B.J. (2021). Sources and reporting of oil spills and impacts on wildlife 1970–2018. Environ. Sci. Pollut. Res..

[29] Henkel L.A., Ziccardi M.H. (2018). Life and death: How should we respond to oiled wildlife?. J. Fish Wildl. Manag..

[30] Helm R.C., Carter H.R., Ford R.G., Fry D.M., Moreno R.L., Sanpera C., Tseng F.S., Fingas M. (2015). Overview of Efforts to Document and Reduce Impacts of Oil Spills on Seabirds. Handbook of Oil Spill Science and Technology.

[31] Short M. Managing Human Risk During an Oiled Wildlife Response. Proceedings of the International Oil Spill Conference Proceedings.

[32] Morgan A.D., Shaw-Brown K., Bellingham I., Lewis A., Pearce M., Pendoley K. Global Oil Spills and Oiled Wildlife Response Effort: Implications for Oil Spill Contingency Planning. Proceedings of the International Oil Spill Conference Proceedings.

[33] Clumpner C., Callahan B. Optimizing the Value of Near Misses in Wildlife Response Preparedness: The Kulluk Incident. Proceedings of the International Oil Spill Conference Proceedings.

[34] Gartrell B.D., Battley P.F., Clumpner C., Dwyer W., Hunter S., Jensen M., McConnell H.M., Michael S., Morgan K.J., Nijman P. (2019). Captive husbandry and veterinary care of seabirds during the MV Rena oil spill response. Wildl. Res..

[35] Sharp B.E. (1996). Post-release survival of oiled, cleaned seabirds in North America. Ibis.

[36] Reeve C.L., Spitzmuller C., Rogelberg S.G., Walker A., Schultz L., Clark O. (2004). Employee reactions and adjustment to euthanasia-related work: Identifying turning-point events through retrospective narratives. J. Appl. Anim. Welf. Sci..

[37] Rohlf V., Bennett P. (2005). Perpetration-induced traumatic stress in persons who euthanize nonhuman animals in surgeries, animal shelters, and laboratories. Soc. Anim..

[38] Scotney R.L., McLaughlin D., Keates H.L. (2015). A systematic review of the effects of euthanasia and occupational stress in personnel working with animals in animals shelters, veterinary clinics, and biomedical research facilities. J. Am. Vet. Med. Assoc..

[39] Chilvers B.L., Finlayson G., Ashwell D., Low S.I., Morgan K.J., Pearson H.E. (2016). Is the way an oil spill response is reported in the media important for the final perception of the clean-up?. Mar. Pollut. Bull..

[40] Nijkamp H., Sessions S., Blanc P., Autret Y. Artic Oiled Wildlife Response: Exploring Potential and Limitations. Proceedings of the International Oil Spill Conference Proceedings.

[41] Hunt S., Smith K., Hamerton H., Sargisson R.J. (2014). An incident control centre in action: Response to the Rena oil spill in New Zealand. JCCM.

[42] Smith K., Hamerton H., Hunt S., Sargisson R.J. (2016). Local volunteers respond to the Rena oil spill in Maketu, New Zealand. Kotuitui N. Z. J. Socl. Sci. Online.

[43] Spears R.E., Helton S.E., Pease A.L., Wendel T.R. Volunteers at Oil Spill Clean-Ups: Guidance for On-Scene Coordinator. Proceedings of the International Oil Spill Conference Proceedings.

[44] Clumpner C. Personnel for Oiled Wildlife Response: Challenges in Identifying, Training, and Maintaining an Effective Team. Proceedings of the International Oil Spill Conference Proceedings.

[45] Hur J.Y. (2012). Disaster management from the perspective of governance: Case study of the Hebei Spirit oil spill. Disaster Prev. Manag..

[46] Yeung P., White B., Chilvers B.L. (2017). Exploring wellness of wildlife carers in New Zealand: A descriptive study. Anthrozoos.

[47] Gibson D. Worker Stress and Fatigue in Oil Spill Response. Proceedings of the International Conference on Health, Safety and Environment in Oil and Gas Exploration and Production.

[48] Fowler H.N., Holzbauer S.M., Smith K.E., Scheftel J.M. (2016). Survey of occupational hazards in Minnesota veterinary practices in 2012. J. Am. Vet. Med. Assoc..

[49] White B., Yeung P., Chilvers B.L., O’Donoghue K. (2021). Reducing the “cost of caring” in animal-care professionals: Social work contribution in a pilot education program to address burnout and compassion fatigue. J. Hum. Behav. Soc. Environ..

[50] Figley C.R. (1995). Compassion Fatigue: Coping with Secondary Traumatic Stress Disorder in Those who Treat the Traumatized.

[51] Brady J.L., Guy J.D., Poelstra P.L., Brokaw B.F. (1999). Vicarious traumatization, spirituality, and the treatment of sexual abuse survivors: A national survey of women psychotherapists. Prof. Psychol. Res. Pract..

[52] Stamm B.H. (2010). The Concise ProQOL Manual: The Concise Manual for the Professional Quality of Life Scale.

[53] Smart D., English A., James J., Wilson M., Daratha K.B., Childers B., Magera C. (2014). Compassion fatigue and satisfaction: A cross-sectional survey among US healthcare workers. Nurs. Health Sci..

[54] Burnett H., Wahl K. (2015). The compassion fatigue and resilience connection: A survey of resilience, compassion fatigue, burnout, and compassion satisfaction among trauma responders. Int. J. Emerg. Ment. Health Hum. Resil..

[55] Gonzalez T.C., Burnett H.J., Helm H., Edwards L. (2019). An examination of resilience, compassion fatigue, burnout, and compassion satisfaction between men and women among trauma responders. N. Am. J. Psychol..

[56] Wagnild G., Young H. (1993). Development and psychometric evaluation of the Resilience Scale. J. Nurs. Manag..

[57] Aiena B.J., Buchanan E.M., Smith C.V., Schulenberg S.E. (2016). Meaning, resilience, and traumatic stress after the Deepwater Horizon oil spill: A study of Mississippi coastal residents seeking mental health services. J. Clin. Psychol..

[58] Smith B.W., Dalen J., Wiggins K., Tooley E., Christopher P., Bernard J. (2008). The brief resilience scale: Assessing the ability to bounce back. Int. J. Behav. Med..

[59] Judge T.A., Locke E.A., Durham C.C., Kluger A.N. Brief Job Satisfaction Measure II. http://www.timothy-judge.com/JS2.htm.

[60] Warr P., Cook J.D., Wall T.D. (1979). Scales for the measurement of work attitudes and psychological wellbeing. J. Occup. Organ. Psychol..

[61] Quinn R., Staines G. (1979). The 1972-73 Quality of Employment Survey: Descriptive Statistics, with Comparison Data from the 1969-70 Survey of Working Conditions.

[62] Kidd A.H., Kidd R.M., Zasloff R.L. (1996). Characteristics and motives of volunteers in wildlife rehabilitation. Pscychol. Rep..

[63] Kranke D., Weiss E., Heslin K., Dobalian A. (2017). “We are disaster response experts”: A qualitative study on the mental health impact of volunteering in disaster settings among combat veterans. Soc. Work Public Health.

[64] Tabassum F., Mohan J., Smith P. (2016). Association of volunteering with mental well-being: A lifecourse analysis of a national population-based longitudinal study in the UK. BMJ Open.

[65] Gabassi P.G., Cervai S., Rozbowsky P., Semeraro A., Gregori D. (2002). Burnout syndrome in the helping professions. Psychol. Rep..

[66] Maslach C., Jackson S.E. (1981). The measurement of experienced burnout. J. Organ. Behav..

[67] Burnett H. (2017). Revisiting the compassion fatigue and resilience connection: A survey of resilience, compassion fatigue, burnout, and compassion satisfaction among trauma responders. J. Police Emerg. Resp..

[68] Fan Y., Guan L., Xiang H., Yang X., Huang G., Cheng W., Xie Y., Wang X., Liang G., He M. (2021). A longitudinal study on emotional distress among local government staff seven years after the 2008 Wenchuan earthquake in China. BMC Public Health.

[69] Lopez-lbor J.J. (2006). Disasters and mental health: New challenges for the psychiatric profession. World J. Biol. Psychiatry.

[70] Prati G., Pietrantoni L., Cicognani E. (2010). Self-efficacy moderates the relationship between stress appraisal and quality of life among rescue workers. Anxiety Stress Coping.

[71] Enarson E. (2001). What women do: Gendered labor in the Red River Valley flood. Glob. Environ. Chang. Part B Environ. Haz..

[72] Enarson E. (2012). Women Confronting Natural Disaster: From Vulnerability to Resistance.

[73] Dhungel R., Ojha R.N. (2012). Women’s empowerment for disaster risk reduction and emergency response in Nepal. Gend. Dev..

[74] Aslan H., Erci B., Pekince H. (2021). Relationship between compassion fatigue in nurses, and work-related stress and the meaning of life. J. Relig. Health.

[75] Gleichgerrcht E., Decety J. (2013). Empathy in clinical practice: How individual dispositions, gender, and experience moderate empathic concern, burnout, and emotional distress in physicians. PLoS ONE.

[76] Mooney C., Fetter K., Gross B.W., Rinehart C., Lynch C., Rogers F.B. (2017). A preliminary analysis of compassion satisfaction and compassion fatigue with considerations for nursing unit specialization and demographic factors. J. Trauma Nurs..

[77] Atkins C.D., Burnett H.J. (2016). Specialized disaster behavioural health training: Its connection with response, practice, trauma health, and resilience. Disaster Health.

[78] Pollock C., Paton D., Smith L., Violanti J., Paton D., Violanti J.M., Smith L.M., Charles C. (2003). Training for Resilience. Promoting Capabilities to Manage Posttraumatic Stress: Perspectives on Resilience.

[79] Schiraldi G.R., Jackson T.L., Brown S.L., Jordan J.B. (2010). Resilience training for functioning adults: Program description and preliminary findings from a pilot investigation. Int. J. Emerg. Ment. Health.

[80] Mealer M., Jones J., Newman J., McFann K.K., Rothbaum B., Moss M. (2012). The presence of resilience is associated with a healthier psychological profile in intensive care unit (ICU) nurses: Results of a national survey. Int. J. Nurs. Stud..

[81] Ray S.L., Wong C., White D., Heaslip K. (2013). Compassion satisfaction, compassion fatigue, work life conditions, and burnout among frontline mental health care professionals. Traumatology.

[82] Wolfaardt A.C., Williams A.J., Underhill L.G., Crawford R.J.M., Whittington P.A. (2009). Review of the rescue, rehabilitation and restoration of oiled seabirds in South Africa, especially African penguins Spheniscus demersus and Cape gannets Morus capensis, 1983–2005. Afr. J. Mar. Sci..

[83] Siewright K.A., Battley P.F., McConnell H.M., Armstrong D.P., Morgan K.J. (2019). Survival rates of oil-rehabilitated and non-rehabilitated little penguins after the C/V Rena oil spill, New Zealand. Mar. Pollut. Bull..

[84] Siewright K.A., Battley P.F., McConnell H.M., Chilvers B.L., Morgan K.J. (2019). Post-release breeding success of oil-rehabilitated and non-rehabilitated little blue penguins, Eudyptula minor, following the M/V Rena oil spill, New Zealand. Mar. Pollut. Bull..

[85] Chilvers B.L., Battley P.F. (2019). Species prioritization index for oiled wildlife response planning in New Zealand. Mar. Pollut. Bull..

[86] Arora S., Sevdalis N., Nestel D., Woloshynowych M., Darzi A., Kneebone R. (2010). The impact of stress on surgical performance: A systematic review of the literature. Surgery.

[87] Patel V.L., Cohen T., Murarka T., Olsen J., Kagita S., Myneni S., Buchman T., Ghaemmaghami V. (2011). Recovery at the edge of error: Debunking the myth of the infallible expert. J. Biomed. Inf..

[88] Brannick E.M., DeWilde C.A., Frey E., Gluckman T.L., Keen J.L., Larsen M.R., Helke K.L. (2015). Taking stock and making strides toward wellness in the veterinary workplace. J. Am. Vet. Med. Assoc..

